# Oral manifestations of delusional infestation: a case series

**DOI:** 10.1186/s12903-022-02664-7

**Published:** 2022-12-29

**Authors:** Zahra Alsafwani, Morooj Aljishi, Caroline Shiboski, Richard Jordan, Alessandro Villa

**Affiliations:** 1 Department of Orofacial Sciences, School of Dentistry, University of California San Francisco, 513 Parnassus Avenue, S- 722, San Francisco, CA 94143 USA; 2 Department of Biomedical Science, College of Dentistry, Imam Abdulrahman Bin Faisal University, IAU, Dammam, Saudi Arabia; 3 Miami Cancer Institute, Baptist Health South Florida, Miami, FL USA

**Keywords:** Delusional infestation, Delusional parasitosis, Psychiatric disorder, Oral medicine, Case report, Case series

## Abstract

**Background:**

Delusional infestation (DI) is a rare psychotic disorder characterized by a patient’s false belief that the body is infested with living or non-living organisms in the absence of clinical evidence of disease. Based on the underlying etiology, DI can be classified into primary and secondary forms based on the presence or absence of an underlying condition or previously diagnosed psychiatric disorder. This paper discusses a condition that is not commonly reported in the literature.

**Case presentation:**

Here, we describe four patients diagnosed with DI of the oral cavity. In each case, the patients’ intraoral examinations revealed either traumatic lesions or no signs of mucosal disease. Management involved symptom management, medical therapy, and/or a referral to the primary care provider.

**Conclusion:**

Because oral health care providers may encounter patients with DI, they should familiarize themselves with this unusual condition in order to recognize the condition and initiate prompt referral to a psychiatrist.

## Background

Delusional infestation (DI; also called delusional parasitosis) is a rare psychotic disorder characterized by a patient’s false belief that their body is infested by living or non-living organisms in the absence of any clinical evidence of disease [1, 2]. DI is classified into primary and secondary forms: the primary type presents with cutaneous and oral symptoms but no underlying conditions or previous psychiatric disorder [3] and the secondary type is associated with an underlying psychiatric disorder including schizophrenia, depression, dementia, anxiety, and others, or secondary to medication or illicit drug use [2].

The prevalence of DI has been reported to be 6/100,000 patients seen in hospitals and in public health services, and 83/100,000 patients seen in private practice settings [1]. Females are more often affected than males [1]. Patients typically describe symptoms of tingling, itching, crawling, and sensation of movement under the skin and mucosa [2]. Patients attribute these abnormal sensations to the presence of worms, insects, or other organisms, under the skin and mucosa [2]. A phrase that is quite similar to delusional manifestation is referred to as cenesthopathy, which is a feeling of a foreign body without any dental or medical evidence. Cenesthopathy most commonly affects the oral cavity, hence the term “oral cenesthopathy.” Some patients report strange oral sensations like increased mucus production or a slimy feeling, while others report peculiar oral sensations like the perception of coils or wires in the mouth [4]. Individuals with DI may attempt to remove the fictitious pathogens utilizing exfoliators, fingernails or tweezers, and harsh cleansers that may result in excoriations, ulcerations, cuts, and sometimes secondary infections. It is essential to conduct a thorough clinical examination to distinguish DI from other similar appearing diseases [2]. Blood tests may be helpful to rule out uncontrolled diabetes mellitus that may lead to neuropathy and contribute to oral dysesthesias. Drug abuse is also a consideration in this setting [5]. Dental providers may encounter cases of DI during their clinical practice, but the rarity of the condition and absence of clinical findings could pose a diagnostic challenge. Here, we describe four cases of DI presenting with varying oral signs and symptoms so that oral health care providers can familiarize with this condition and the importance of close interdisciplinary collaboration amongst clinicians.

## Case presentation

### Case 1

A 50-year-old female presented to the Oral Medicine Clinic at our institution as self-referred with a chief complaint of parasitic infection that had worsened in the past 18 months. Her past medical history included microcytic iron deficiency anemia, thalassemia, and gastroesophageal reflux disease. She did not report any history of psychiatric disorders. The patient had a history of methamphetamine drug abuse 3 years prior. She reported being in good health until a year and a half earlier, when she developed fatigue and “brain fog” and had a 66-lb (30 kg) unintentional weight loss. After consulting with several physicians, one of whom was an infectious disease specialist, a diagnosis of strongyloides was made due to the presence of protozoa and bacteria nematode in her stool culture. She also reported having a sudden onset of generalized gingival inflammation and dental caries that resulted in the loss of multiple teeth.

**Fig. 1:**
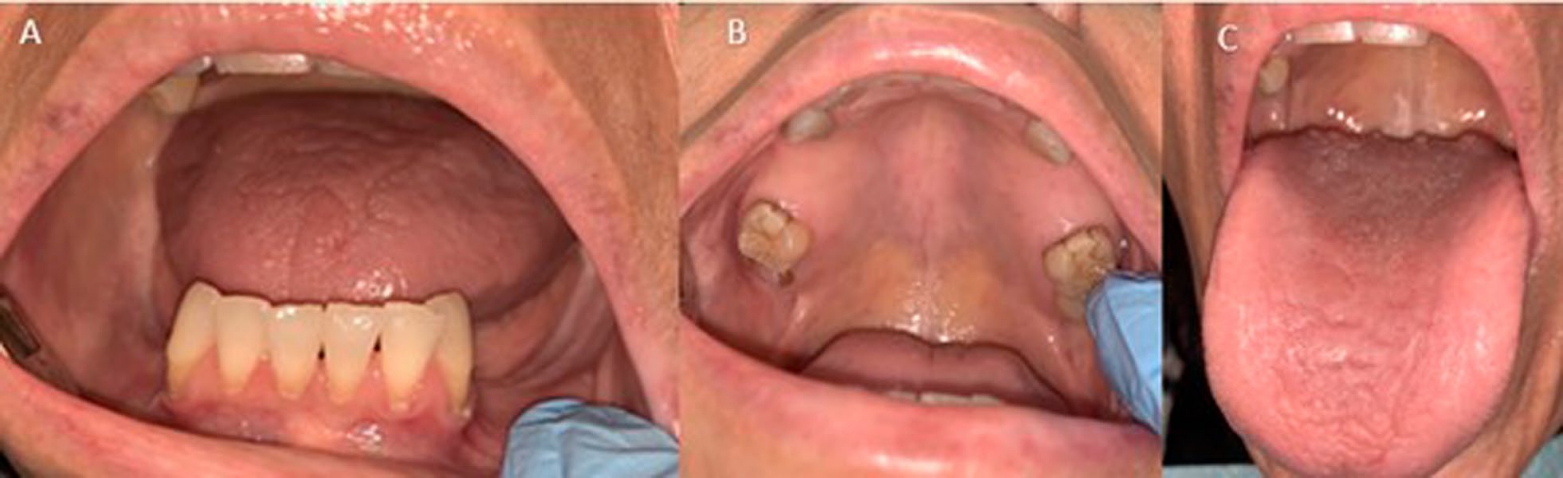
Panel 1**A**: multiple missing mandibular teeth; Panel 1**B**: multiple missing maxillary teeth and a remaining root of tooth #1; Panel 1**C**: depapillated tongue dorsum

At the time of presentation, the patient self-reported having multiple “parasites and worms” under her gingivae and dental crowns. During the visit she produced several pictures on her smartphone of what appeared to be normal saliva. However, she insisted that her saliva contained multiple microorganisms. Intraoral examination revealed generalized dry mouth, de-papillated tongue dorsum, and multiple missing maxillary and mandibular teeth (Fig. 1; panels 1A–C). There were no clinical signs of fungal, bacterial or viral infection. The patient was advised to increase hydration to 2 L of water daily, and pilocarpine HCl 5 mg three times a day was prescribed for her dry mouth. Based on the patient’s chief complaint (she reported “having parasites” in her mouth), and clinical presentation, a provisional diagnosis of DI was made, and she was referred to her primary care physician and infectious disease specialist for further evaluation and management. The patient never returned for follow up care.

### Case 2

A 43-year-old male presented for a video consultation with a chief complaint of worsening painful multiple oral lesions with a reported pain score of 10/10. The oral lesions had developed 6 weeks prior, and he described having developed a “natural” regimen to control his pain consisting in rinsing his mouth with hydrogen peroxide multiple times a day and brushing his tongue with a manual and with an electric toothbrush for 5 min each time multiple times a day to “get rid of the mouth infection.” The patient had no history of psychiatric disorders. His past medical history included *Staphylococcus aureus* infection when he was 2 years old (per patient’s report) and multiple recurrent episodes of skin infection that he thought were also caused by *S. aureus*. He reported a recent visit to his primary care provider for skin rash in which she thought that he had another flare up of his *S. aureus* skin infection and prescribed a long course of cefalexin 500 mg twice a day. She also advised him to continue mupirocin topical applications twice/day. His social history revealed that he never smoked cigarettes although used therapeutic marijuana and drank alcohol only very occasionally.

Extraoral examination performed using video-conferencing revealed a maculopapular rash of the neck and around his right ear. The patient provided several intraoral photographs prior to the visit showing multiple large oral ulcers of the buccal mucosa bilaterally, right retromolar trigone hard palatal mucosa and lower right buccal vestibule Based on his description and our limited video-examination, it was suspected that the patient also had an area of exposed bone in the left mylohyoid ridge area. During the video visit the patient insisted he had an “active *S. aureus* infection” in his mouth. He was prescribed chlorhexidine 0.12% rinse twice a day and 2% viscous lidocaine for the oral pain and an in-person visit was scheduled for an incisional biopsy and full workup shortly thereafter. He was advised to discontinue the hydrogen peroxide solution and was also referred to the Dermatology and Infectious disease services for assessment.

**Fig. 2:**
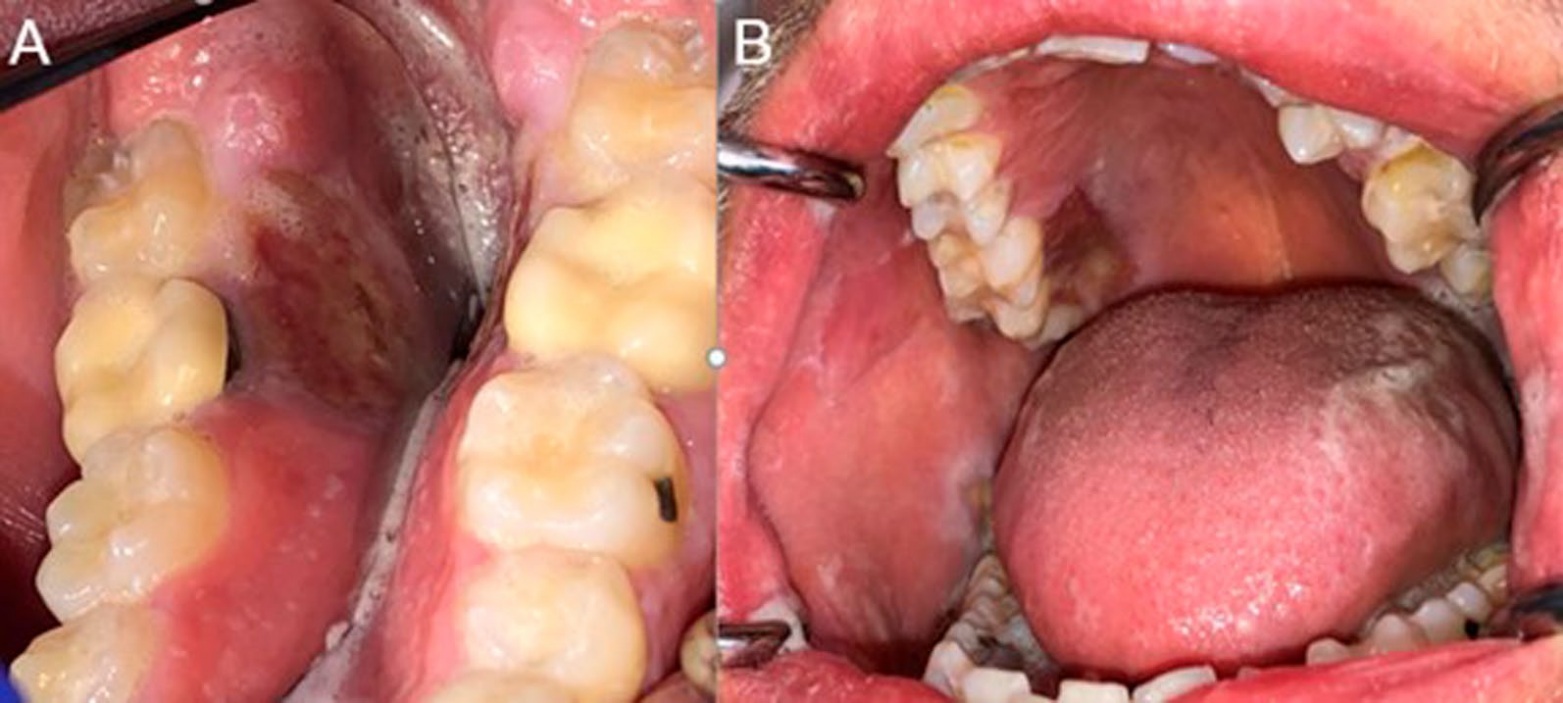
Panel **A**: large erosions and ulcers of the left mylohyoid ridge. Panel **B**: Ulcers of the posterior right palatal and buccal mucosa, and left ventral tongue

**Fig. 3:**
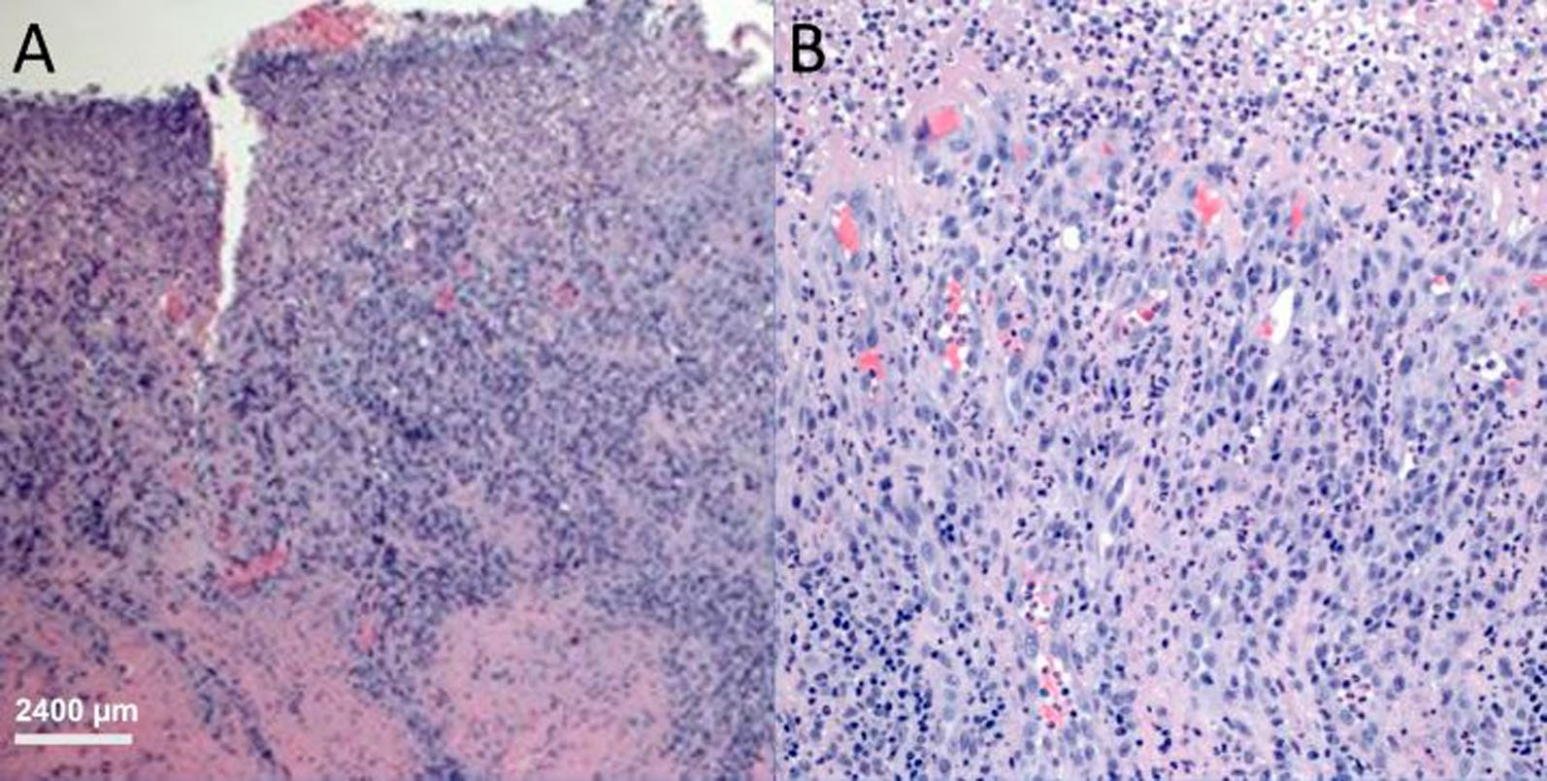
Panel **A** (H&E) incisional biopsy of right buccal mucosa sections show mucosa that is mainly ulcerated with a base formed by inflamed granulation tissue and covered by fibrinous exudate; Panel **B**: The infiltrate at the base is comprised of lymphocytes, plasma cell and neutrophils. 4 × objective was used on an OLYMPUS BX53 microscope at 40 × magnification. Camera was Infinity 3 by Teledyne.Software used was Teledyne Lumera Infinity analyze version 7.0.3.1111

One week later, the patient presented for an in person visit and reported similar oral symptoms with a pain score of 8–9/10. He reported brushing his teeth and gingiva after each meal for about two hours and started rinsing with chlorhexidine “straight out of the bottle” three times per day (after each meal). He brought several zip-lock bags with what appeared to be bone fragments, although he described the content of the bags as foreign bodies and bacteria that he had to remove from his mouth. He reported that the sores usually healed once he removed all the foreign bodies/bacteria. Oral examination revealed several large erosions and ulcers on the right buccal mucosa and hard palate, and on the left mylohyoid ridge (Fig. 2. Panels A and B), although the lesions were less extensive than on the pictures the patient provided during his video visit. Our differential diagnosis included self-induced injuries, and a biopsy was recommended to rule out any other pathological processes including infection. An incisional biopsy of the right buccal mucosa was performed and revealed a “nonspecific ulcer with no evidence of candidiasis, herpetic viral cytopathic effect, dysplasia or malignancy” (Fig. 3). The results of the biopsy were discussed with the patient, who continued to insist he had an oral bacterial infection and denied the possibility that the ulcerations were self-induced. The patient history and behaviors, clinical features, and biopsy results strongly suggested a diagnosis of DI.

The infectious disease specialist, who the patient subsequently consulted upon our recommendation, found no evidence of Methicillin-resistant *S. aureus* infection (MRSA) and recommended a psychiatric evaluation. Two weeks later the patient reported by email that he had developed lip swelling with continued oral pain and discomfort. The patient refused to have a follow up video visit and requested a second biopsy. When a referral to the primary care provider was recommended the patient canceled his appointment and was lost to follow-up.

### Case 3

A 47-year-old male was referred to the Oral Medicine Clinic by an infectious disease specialist for the evaluation of an asymptomatic white tongue lesion. The patient reported that he first noticed the lesion on the left posterior surface of the tongue with frequent “flare-up and remission” episodes 18 months prior to presentation. The patient had a previous history of anal fistula, and a possible diagnosis of Crohn’s disease and syphilis was made after seeing several specialists for it. Per his report, further investigations by an infectious disease specialist were negative for syphilis; these included blood tests for enzyme-linked immunosorbent assay (ELISA), rapid plasma regain (RPR) and by lumbar puncture. He was also tested for *Giardia duodenalis* infection with negative results as well. As part of the social history patient reported that he was homosexual. There was no history of psychiatric conditions. During the intraoral examination, there was a well demarcated patchy erythematous lesion on the left lateral border of the tongue surrounded by a white rim, measuring around 1 cm in diameter; a diagnosis of geographic tongue was made. The findings and diagnosis were explained to the patient, but he refused to accept it and believed that his condition was misdiagnosed. Despite all the assessments, the patient continued to believe he had an active oral syphilitic infection. Patient was then referred to an infectious disease specialist for further evaluation and management. All tests were inconsistent with syphilis or neurosyphilis, however the patient remain convinced that he had syphilis. Finally, the patient decided to see an outside provider in a local community Hospital who obtained a tongue biopsy which revealed “benign squamous mucosa with acute and chronic inflammation and reactive changes.” He never returned to the Oral Medicine Clinic. Based on the patient’s symptoms, clinical picture of “geographic tongue”, infectious disease specialist evaluation and tongue biopsy, a diagnosis of DI was made.

### Case 4

**Fig. 4:**
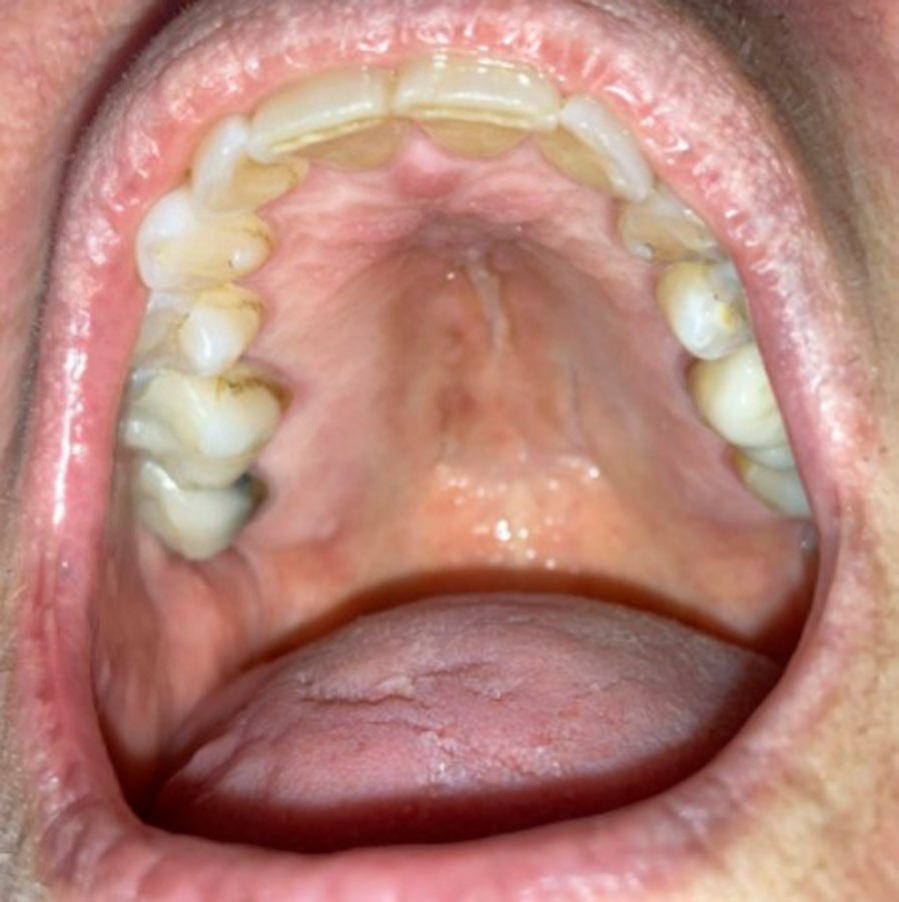
Hard palatal mucosa in a patient with oral delusional infestation with no lesions noticed on clinical exam

A 69-year-old woman was referred to the Oral Medicine Clinic by an otolaryngologist for the evaluation of oral erythema and dryness. Her past medical history was significant for breast cancer status post-surgical treatment, mucormycosis, Herpes simplex virus 2 (HSV-2) and vertigo. She had no history of psychiatric diseases. Extraoral examination was within normal limits. Intraoral examination revealed mild oral dryness and no other signs of mucosal lesions, swelling or infections (Fig. 4). The patient reported that she developed a sore throat and “bumps” on the palatal mucosa after severe acute respiratory syndrome–related coronavirus (SARSr-CoV) infection a few months prior to presentation. The diagnosis of SARSr-CoV infection was not confirmed by testing. She reported applying Lysol^®^ disinfecting wipe (an American brand of cleaning and disinfecting products distributed by Reckitt) inside her mouth multiple times a day for three minutes each time to “clear COVID-19” with self-reported improvement of the oral redness. She also reported having “black lines under her eye” in the past that resolved after applying the Lysol^®^ disinfecting wipes. Patient was instructed to use Biotene^®^ rinses as needed for xerostomia, and to stop using the Lysol^®^ disinfecting wipe inside the mouth. A possible diagnosis of DI was made, and the patient was referred to her primary care physician for further evaluation and management.

## Discussion and conclusion

DI is a rare disorder, and its oral manifestation may represent a diagnostic challenge to the clinician due to its rarity and the frequent absence of clinical findings. Here, we present four cases of DI involving the mouth. None of the patients had signs of oral infections, yet every patient believed they had an active infection and/or microorganisms in the oral cavity. Several terms have been used over the years for DI including delusional parasitosis, Ekbom syndrome, acarophobia, and parasitophobia [1]. The term DI was initially proposed by Freudenmann and Lepping to comprehensively include all the animate or inanimate pathogens the patients claim to be infested with [1]. In some instances, patients have presented with a self-diagnosis of ‘Morgellons syndrome’, a controversial condition initially described by Mary Leitao after she identified thin filaments protruding from wounds around the mouth of her 2-year-old son [6, 7]. Investigators at the Centers for Disease Control and Prevention conducted a study of 115 patients with similar complaints and were not able to determine whether this was a new entity or an extension of DI. No parasites or mycobacteria were detected. Most materials collected from participants’ skin were composed of cellulose, likely of cotton origin [8]. A large retrospective study conducted in Japan in 2015 reported on 606 patients with oral cenesthopathy. Cenesthopathy is a condition in which patients complain of abnormal sensations in a particular area of the body. The study concluded that the majority of patients were elderly females and the dental care received during the acute depressive episode could have been a risk factor for developing cenesthopathy. A careful history and consultation with a psychiatrist may be needed for these patients [4].

Most patients with DI present to a clinician with material collected in a container as evidence of infestation [1, 2]. One of our patients (patient #1) showed pictures of what appeared normal saliva and was convinced it contained parasites and worms. Laboratory examination of these specimens usually reveals a collection of scabs, hair, skin debris, or occasionally, plants or insects, without signs of parasite infestation [1, 7]. Patients #2 and #3 had similar delusional thoughts and both assumed they had an active oral bacterial infection. Patient #2 thought he had active infection with *S. Aureus* since childhood, even though the oral biopsy revealed a non-specific ulceration, and the infectious disease specialist ruled out infection. Patient #3 continued to believe he had syphilis, despite a negative serology, and the oral presentation was more consistent with geographic tongue. Patient #4 reported a possible oral infection from COVID-19 and thought that she still had some possible related side effects including “bumps” in her palate and “dark lines” under her eyes for which she used Lysol disinfecting wipes in her mouth.

There are few cases in the literature reporting oral manifestations of DI. Those are summarized in Table 1 [9–12], in addition to the cases reported in this paper. Comparisons with previous case reports have identified similar patterns of complaints as described in our series. One prior reported case described similar delusional thoughts in a 31 years old-female patient who thought she was infested with lizards, beetles, and crickets; she also reported feelings of guilt that contributed to her delusion. This patient showed improvement with a combination of pimozide and imipramine [3]. Another case report described Morgellons disease in a 39-year-old female who had an initial chief complaint of hair and insects originating from her gingiva causing oral tickling and gingival bleeding. She was instructed to follow up with a psychiatrist [13]. Finally, another report described a 64-year-old woman who believed her oral cavity was infested with numerous live worms, but the patient’s symptoms resolved with amitriptyline [14].

**Table 1:** Summary of previous case reports of delusional infestation with oral manifestation

Author	Sex	Age (year)	Chief complaint	Treatment
Maeda [9]	Male	76	Thread emerging from between his teeth, which later evolved into worms	Trial of pimozide, an antipsychotic agent
Ghaffari [3]	Female	31	Lizards, beetles, and crickets infesting the oral cavity	Combination of pimozide and imipramine
Hanihara [14]	Female	64	Oral cavity infested with numerous live worms	Patient treated successfully with sulpiride and amitriptyline
Dovigi [10]	Female	61	Persistent oral lesion with emergence of numerous fibers over a 2-year period. Experienced itching and irritation in the affected area. Later admitted to scratching the site with her fingernail	Oral biopsy performed, showed synthetic polymer fibers
Grosskopf [13]	Female	39	Hair and insects originating from the gingiva causing oral tickling and gingival bleeding	Patient instructed to follow up with a psychiatrist
Davis [11]	Female	66	Dry mouth and parasitic film coating her teeth	An oral surgeon ruled out infection/parasites and diagnosed her with mild gum disease. She was on anti-parkinsonism medications: ropinirole XL (DI symptoms persisted despite discontinuation), and trihexyphenidyl (discontinued to minimize dry mouth). Quetiapine 25 mg/night was ineffective and was replaced with clonazepam 1 mg/night with resolution of symptoms
Ochiai [12]	Female	89	Oral cenesthopathy, which is a feeling of filament structures in her mouth	Combination of donepezil 5 mg/day and aripiprazole 1.5 mg/day
Our case 1	Female	50	Parasitic infection that had worsened in the past 18 months	Referred to PCP^1^ and an infectious disease specialist for further evaluation
Our case 2	Male	43	Painful multiple oral lesions with a belief of having active *Staphylococcus aureus* infection	Oral biopsy showed no specific ulcer. Referral to the PCP^1^ was recommended
Our case 3	Male	47	White tongue lesion and self-diagnosis of syphilis	Diagnosis of geographic tongue was made
Our case 4	Female	69	Oral erythema and dryness which she believed were caused by SARS-CoV-22^2^, although not confirmed by a test. She used Lysol^®^ disinfecting wipe intraorally to clear the infection	Patient was referred to her PCP^1^ for further evaluation and management

1: Primary care physician

2: Severe acute respiratory syndrome coronavirus 2

When encountered in the dental office, patients with a possible diagnosis of DI should be referred for a psychiatric evaluation [13]. Treatment for patients diagnosed with DI and mild depressive symptoms is cognitive behavioral therapy (CBT) prior to pharmacological treatment [15]. The goal of CBT is to establish trust and develop a connection between a patient’s feelings, emotions, and behaviors which will lead to significant improvement in the patient’s social life [15]. In patients with moderate depressive symptoms, antidepressants may be needed. Selective serotonin reuptake inhibitors (SSRI) are considered the first line of treatment [2].A combination with antipsychotics has been shown to lead to the less side effects [2]. Electroconvulsive therapy (ECT) might be considered in patients with delusional parasitosis secondary to medication-resistant depression [2].

Of interest, all the oral DI cases we reported were observed during the recent COVID-19 pandemic over a period of less than 12 months, which seems like a much higher frequency than what we have encountered previously during the many years of our practice. As previously mentioned, DI is an uncommon condition, making our observation an unusual one. The pandemic has greatly impacted the lives of many people, and in particular, individuals who suffer from mental disorders. These patients depend on their health care providers or their support network for psychiatric treatment. All of these social interactions were either restricted or limited during the pandemic to prevent the spread of COVID-19 [16]. Interestingly, Sukut et al. mentioned that isolation and quarantine can cause patients to experience dread, worry, and uncertainty, resulting in an increase of stress-related diseases and exacerbation of pre-existing mental disorders; this eventually leads to the relapse and worsening of their condition [17]. Relapse in mental illness can lead to poor hygiene, inability to implement social distancing or other preventive precaution, failure to notify or seek medical help in a timely manner, and non-adherence to expected therapy [17]. We believe the pandemic may have served as a potential trigger for the patient’s delusional thoughts or as an exacerbating factor for the condition. This case series highlights a range of clinical presentations for patients with oral DI and summarizes other cases reported in the literature. However, a formal diagnosis of psychiatric disorders has not been established by a psychiatrist yet, which is considered a limitation.

Although rare, oral health care providers may encounter cases of delusional infestation, and their familiarity with the condition is crucial to initiate a proper management. In this series we presented four cases with various oral manifestations that can serve as a reference for clinicians when they suspect a diagnosis of DI.

## Data Availability

The datasets generated and/or analyzed during the current study are not publicly available due the privacy and secure patients’ information but are available from the corresponding author on reasonable request and can be provided in a de-identified manner.

## Abbreviations

DI: Delusional infestation
HCL: Hydrogen chloride
MRSA: Methicillin-resistant *Staphylococcus aureus*
ELISA: Enzyme-linked immunosorbent assay
RPR: Rapid plasma regain
HSV-2: Herpes simplex virus 2
SARSr-CoV: Severe acute respiratory syndrome–related coronavirus
COVID-19: Coronavirus disease 2019
CBT: Cognitive behavioral therapy
SSRI: Selective serotonin reuptake inhibitors
ECT: Electroconvulsive therapy
